# Endocrown: A Minimally Invasive Alternative to Conventional Fixed Prosthesis

**DOI:** 10.7759/cureus.74514

**Published:** 2024-11-26

**Authors:** M Kirthiga, George Thomas, Sunil Jose, Arun Shyam, Aparna M

**Affiliations:** 1 Conservative Dentistry and Endodontics, Mahe Institute of Dental Sciences and Hospital, Mahe, IND

**Keywords:** endocrown, endodontically treated teeth, lithium disilicate glass-ceramic, monoblock, post-endodontic restoration

## Abstract

Endodontically treated teeth with compromised coronal tooth structure often require core build-ups with the support of radicular posts. In certain cases, the traditional post and core approach may not be possible due to various anatomical and clinical contraindications. Such cases require more meticulous planning and alternative treatment approaches for successful outcomes. The endocrown technique offers a conservative and minimally invasive approach, providing functional and aesthetic rehabilitation while preserving tooth structure. In this clinical case report, we discuss an endocrown-type restoration made from lithium disilicate ceramic in a maxillary first molar with extensive coronal destruction.

## Introduction

The art and science of endodontics have paved the way for the human species to retain their compromised dentition much longer, thereby contributing to the general health and wellness of the population. Even though successful endodontic treatment helps to preserve natural teeth, the complexities involved in the treatment process may make endodontically treated teeth weaker and more susceptible to fracture. Therefore, post-endodontic management becomes a critical aspect of maintaining the longevity and functionality of such teeth.

The type of post-endodontic management depends on various factors, such as remaining tooth structure, aesthetics, functionality, and economics. Traditional methods of rehabilitation include core build-ups, post and core build-ups, and fixed prostheses such as full crowns. However, these traditional methods can weaken the remaining tooth structure, further leading to less-than-optimal results [1].

The new era heralded the use of conservative and less intrusive restorative techniques such as onlays and overlays to restore endodontically treated teeth. These techniques utilize the entire pulp chamber space as a retentive core, creating a 'monoblock'. This concept led to the innovative technique called 'endocrown'. Pissis created the first endocrown in 1995 [2,3].

Endocrowns offer several advantages over traditional posts and cores with crowns. They are easier to prepare and apply; require fewer clinical visits; provide excellent aesthetics; offer better bonding to dental tissues, thereby minimizing the chances of microleakage; and enhance the overall efficacy of endodontic treatment [4]. This case report describes the successful management of a tooth with a severely compromised structure using the endodontic treatment and an endocrown as post-endodontic rehabilitation.

## Case presentation

**Figure 1:**
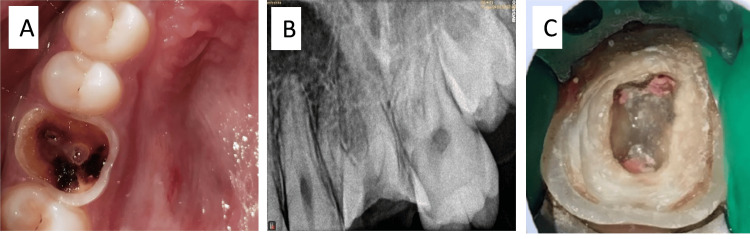
(A) Preoperative clinical image. (B) Preoperative radiograph. (C) After obturation.

A 13-year-old male patient reported a chief complaint of decay in the left upper first maxillary molar. Clinical examination revealed that the tooth had suffered significant damage to its coronal structure due to caries. Based on the available clinical (Figure 1A) and radiographic findings (Figure 1B), the tooth was categorized as badly mutilated, and the treatment options considered included unconventional and complex modalities. There was insufficient crown height with compromised interocclusal space, negating the use of conventional post, core, and crown techniques. We, therefore, narrowed the treatment plan down to a root canal (Figure 1C) followed by IPS-e.Max Press endocrown fabrication and cementation.

Endocrown preparation

**Figure 2:**
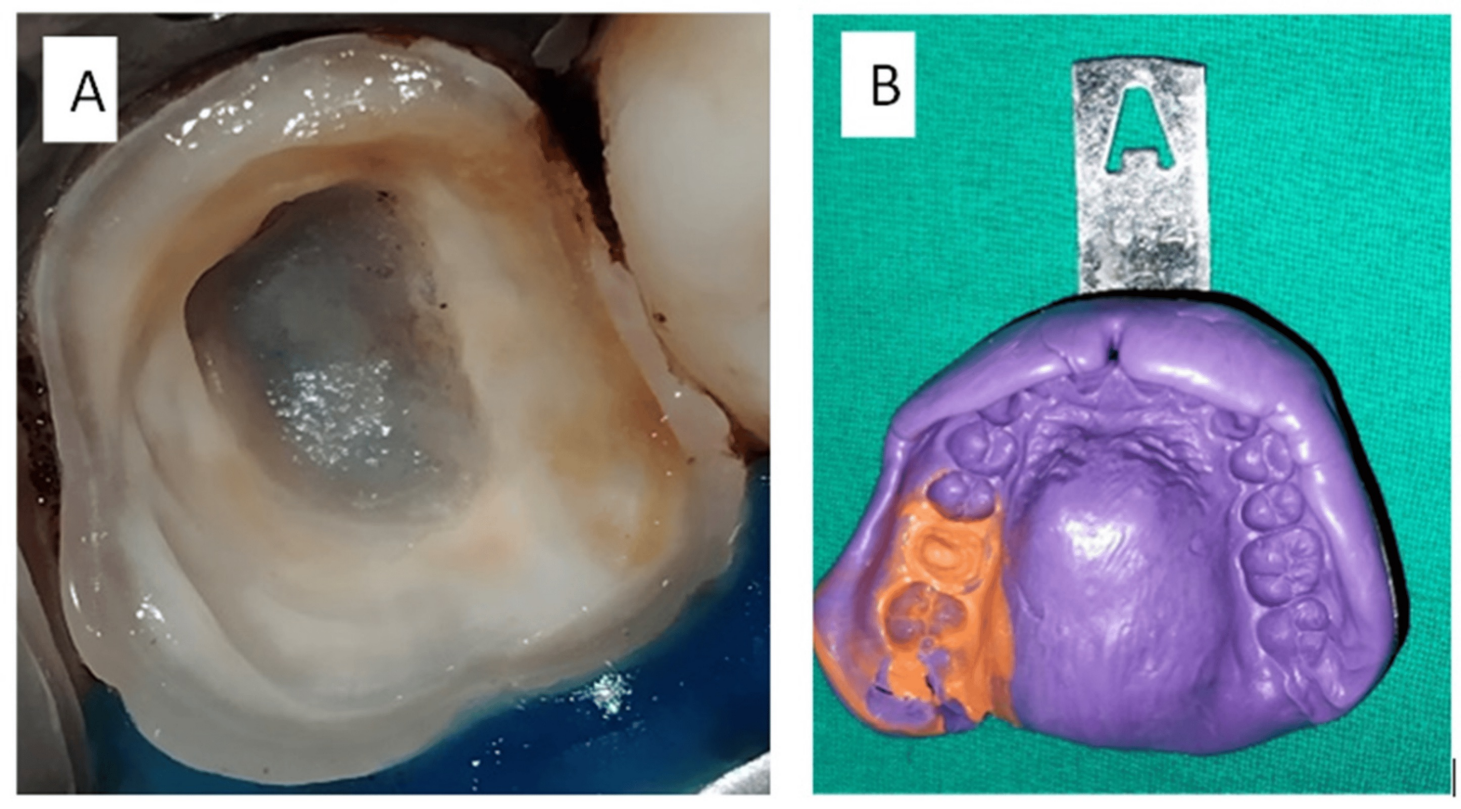
(A) Final endocrown preparation. (B) Putty impression.

We initiated endocrown preparation by removing the gutta-percha not more than 2 mm from the canal orifice. The orifice was subsequently sealed using resin-modified glass ionomer cement (Fuji II LC, GC Corporation, Tokyo, Japan) to ensure the creation of a flat pulpal floor without any undercuts. A coronal reduction of tooth structure was successfully achieved up to 2 mm utilizing a diamond wheel bur (WR-13, Dia-Burs, Mani, Tochogi, Japan). All margins were prepared at an angle of 90 degrees or in a butt joint configuration. A central retentive cavity was established using a diamond-coated bur with coarse grit, with a preparation depth of approximately 4 mm, measured from the pulp chamber roof to the cavosurface margin. Axial preparation was performed with a conical diamond bur featuring a convergence angle of 7 degrees, thereby creating a continuous pathway connecting the coronal pulp chamber to the endodontic access cavity. The cervical margins of the preparation were positioned supragingivally (Figure 2A). Shade was selected using the Vitapan shade guide (A2 shade). The polyvinyl siloxane impression (Flexceed, GC Corporation, India) was recorded employing the putty-wash technique (Figure 2B).

**Figure 3:**
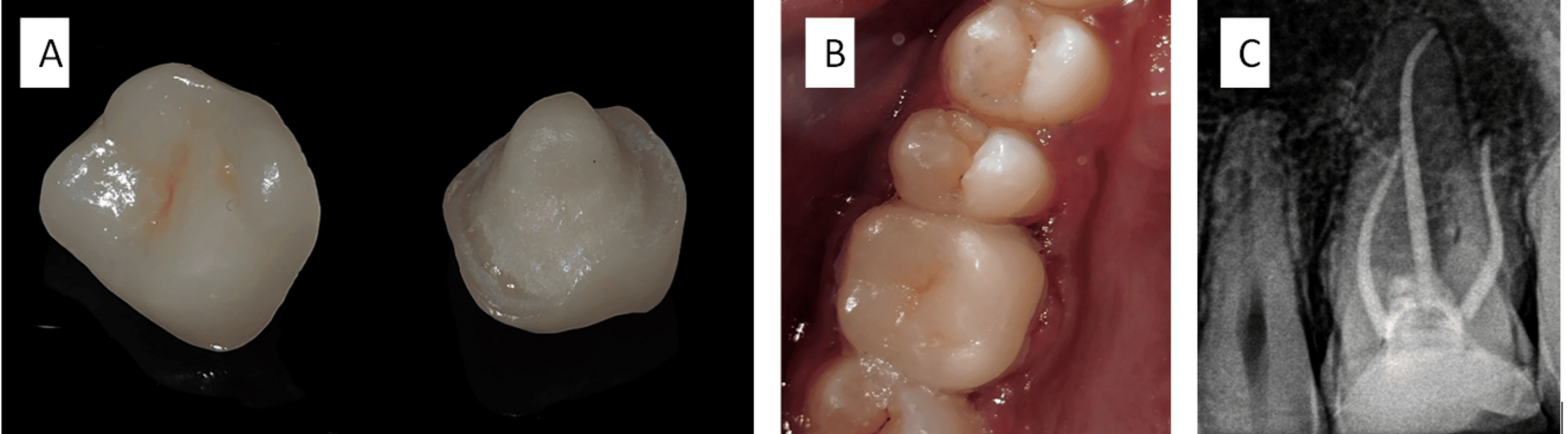
(A) Fabricated endocrown. (B) Clinical photograph showing the cemented endocrown with tooth number 16. (C) Radiograph following the cementation of the endocrown with tooth number 16.

The impression was subsequently sent to the laboratory to fabricate the prosthesis. A provisional acrylic resin restoration was constructed and secured using temporary luting cement. The prosthesis was created utilizing an IPS-e.Max Lithium-disilicate glass-ceramic ingot (Ivoclar Vivadent, Schaan, Liechtenstein). After receiving the prosthesis from the lab (Figure 3A), a try-in was conducted to assess the proper adaptation of the prosthesis. The cementation procedure commenced with etching the internal surface of the prosthesis using 9.6% hydrofluoric acid (Maarc Dental, Maharashtra, India), followed by a thorough rinsing with water and drying with an air syringe. Subsequently, a silane coupling agent was applied for approximately one minute, after which the area was dried. Under rubber dam isolation, we treated the cavity with 37% phosphoric acid (Maarc Dental, Maharastra, India) for 15-20 seconds. We then thoroughly washed and dried the cavity followed by applying single-bond Universal Adhesive (3M ESPE, Neuss-Germany). We implemented light curing for about 20 seconds followed by cementation of the prosthesis using RelyX U200 self-adhesive universal resin cement (3M ESPE, St. Paul, USA) (Figure 3B). A postoperative radiograph confirmed the fit and marginal adaptation of the prosthesis (Figure 3C).

## Discussion

The concept of the endocrown emerged from the idea of combining a minimally invasive treatment approach and the advantages of dentin bonding techniques. Endocrowns also utilize the shape of the pulp chamber as a retentive method [5]. Endocrowns can be considered a version of overlays. Traditional methods of post-endodontic buildups involve periradicular post and core build-ups, which are invasive and compromise the overall resistance of the tooth. The ideal treatment approach is to find a balance between retention and resistance forms. Endocrowns in a way offer this balance in treatment approach.

An endocrown is regarded as a suitable treatment option for all molars, particularly in instances involving short clinical crowns, calcified root canals, or narrow canals. However, it is not considered ideal if adhesion cannot be assured, if the pulpal chamber depth is less than 3 mm, or if the cervical edge is narrower than 2 mm for the majority of its circumference [6]. Sedrez-Porto et al. (2016) suggested that endocrowns demonstrate performance that is either comparable to or superior to that of conventional treatment modalities, which include intraradicular posts, direct composite resin, and inlay/onlay restorations [7]. The endocrown preparation technique involves the creation of a circumferential butt margin, as well as the development of a central retention cavity within the pulp chamber. The depth of the butt margin is maintained within a range of 1.0-1.2 mm to achieve optimal efficacy. This creates a single-unit monoblock structure that serves as both the crown and core, without relying on support from the root canals [8]. The retention and stability of dental restorations are closely linked to the shape of the pulpal chamber cavity. The trapezoidal shape of the pulpal chamber cavity in mandibular molars, along with the triangular configuration of maxillary molars, enhances the stability of the restoration, thereby eliminating the need for any additional preparation. Additionally, the saddle form of the pulpal floor further contributes to the restoration's overall stability [9].

In this case, we used lithium disilicate, reinforced glass ceramic (IPS-e.Max Press), for the fabrication of the endocrown. This material has favorable mechanical properties, excellent aesthetic outcomes, and the ability to bond to the tooth structure [10]. According to Biacchi et al. (2012), lithium disilicate-reinforced ceramic endocrowns exhibit a greater degree of fracture resistance when compared to conventional crowns supported by glass fiber posts [11]. Turkistani et al. (2020) conducted a study to assess the fracture resistance of teeth restored with endocrowns. The findings indicate that an increase in the thickness of the endocrown may adversely affect the fracture resistance of the restored teeth [12]. Endocrowns, which have a greater occlusal thickness of 3-7 mm, have been shown to exhibit a significantly higher degree of fracture resistance than conventional crowns, which typically have a thickness of 1.5-2 mm [13]. Kassis et al. (2021) compared the fracture strength of teeth restored with inlays, onlays, and endocrowns and found that endocrowns exhibited high fracture resistance [14].

El Ghoul et al. (2019) conducted a comparative analysis of hybrid ceramic, fiber composite, lithium disilicate, and zirconia-reinforced lithium disilicate endocrowns to evaluate internal and external discrepancies and found that lithium disilicate glass-ceramic exhibited superior fracture resistance under both axial and lateral loading conditions [15]. According to Belleflamme et al. (2017), endocrowns are an effective restoration option for severely compromised molars and premolars. This is especially relevant in circumstances characterized by substantial loss of coronal tissue or the presence of occlusal risk factors, such as bruxism or unfavorable occlusal relationships [16].

This clinical case illustrates the advantages of using a monoblock concept through the fabrication and cementation of an endocrown. Endocrowns can be a very good minimally invasive alternative to traditional methods of restoring badly mutilated teeth. If cost is not a factor, the indications of endocrowns far outweigh their disadvantages. An endocrown is a classic example of technological brilliance in minimally invasive dentistry.

## Conclusions

Endocrowns are a promising treatment option for endodontically treated molars. Compared with traditional methods, endocrowns offer superior aesthetics and mechanical performance while preserving the remaining tooth structure. Therefore, clinicians must consider endocrowns as a viable option in routine restorative practice.
